# Assessing the exposure risk and impacts of pharmaceuticals in the environment on individuals and ecosystems

**DOI:** 10.1098/rsbl.2013.0492

**Published:** 2013-08-23

**Authors:** Kathryn E. Arnold, Alistair B. A. Boxall, A. Ross Brown, Richard J. Cuthbert, Sally Gaw, Thomas H. Hutchinson, Susan Jobling, Judith C. Madden, Chris D. Metcalfe, Vinny Naidoo, Richard F. Shore, Judit E. Smits, Mark A. Taggart, Helen M. Thompson

**Affiliations:** 1 Environment Department, University of York, York, UK; 2 AstraZeneca, Brixham Environmental Laboratory, Brixham, UK; 3 College of Life and Environmental Sciences, University of Exeter, Exeter, UK; 4 Royal Society for the Protection of Birds, Sandy, UK; 5 Department of Chemistry, University of Canterbury, Christchurch, New Zealand; 6 Centre for Environment, Fisheries and Aquaculture Science, Weymouth, UK; 7 Institute for the Environment, Brunel University, Uxbridge, UK; 8 School of Pharmacy and Biomolecular Sciences, Liverpool John Moores University, Liverpool, UK; 9 Institute for Watershed Science, Trent University, Peterborough, Canada; 10 Biomedical Research Centre, University of Pretoria, Pretoria, South Africa; 11 Centre for Ecology and Hydrology, Lancaster, UK; 12 Faculty of Veterinary Medicine, University of Calgary, Calgary, Canada; 13 University of the Highlands and Islands, Thurso, UK; 14 Food and Environment Research Agency, Sand Hutton, UK

**Keywords:** wildlife, endocrine-disrupting chemicals, non-steroidal anti-inflammatory drugs, vultures, risk prediction, bioindicators

## Abstract

The use of human and veterinary pharmaceuticals is increasing. Over the past decade, there has been a proliferation of research into potential environmental impacts of pharmaceuticals in the environment. A Royal Society-supported seminar brought together experts from diverse scientific fields to discuss the risks posed by pharmaceuticals to wildlife. Recent analytical advances have revealed that pharmaceuticals are entering habitats via water, sewage, manure and animal carcases, and dispersing through food chains. Pharmaceuticals are designed to alter physiology at low doses and so can be particularly potent contaminants. The near extinction of Asian vultures following exposure to diclofenac is the key example where exposure to a pharmaceutical caused a population-level impact on non-target wildlife. However, more subtle changes to behaviour and physiology are rarely studied and poorly understood. Grand challenges for the future include developing more realistic exposure assessments for wildlife, assessing the impacts of mixtures of pharmaceuticals in combination with other environmental stressors and estimating the risks from pharmaceutical manufacturing and usage in developing countries. We concluded that an integration of diverse approaches is required to predict ‘unexpected’ risks; specifically, ecologically relevant, often long-term and non-lethal, consequences of pharmaceuticals in the environment for wildlife and ecosystems.

## 1. Introduction

The continued expansion of the human population is leading to escalating demand for resources, including human and veterinary pharmaceuticals. Pressures are exerted by increasingly intensive agriculture and exacerbated by rising human longevity and obesity, leading to more health problems [1]. With this comes a proliferation in the quantity and diversity of pharmaceuticals consumed and subsequently excreted. While the health benefits of medication are fundamentally important, it is only in the past decade that the potential environmental impacts of pharmaceuticals have begun to be considered in detail [2,3]. At a recent Royal Society-funded Research Fellow International Scientific Seminar, experts from diverse research fields discussed the risks to wildlife posed by pharmaceuticals in the environment. Our conclusions have policy relevance to the ongoing debate in the EU over the prospect of environmental quality standards for specific pharmaceuticals.

Pharmaceuticals are a potentially potent group of chemical contaminants, because they are designed to have biological effects at low concentrations. Given evolutionary conservation across vertebrate taxa, both human and veterinary pharmaceuticals may be predicted to act on many non-target species [4]. It is also now emerging that pharmaceuticals and their biotransformation products are present in a range of habitats, some can bioaccumulate and may have significant, but largely unstudied, consequences for individuals, populations and ecosystems [2,3].

## 2. 17α-ethinyloestradiol and non-steroidal anti-inflammatory drugs: lessons learned and wider implications for wildlife

The two clearest cases of pharmaceuticals affecting wildlife to date involve 17β-oestradiol (E2) and the synthetic oestrogen 17α-ethinyloestradiol (EE2), and the non-steroidal anti-inflammatory drug (NSAID), diclofenac [5,6]. There is now convincing evidence of the feminization of male fish downstream of sewage treatment works (STWs) discharging complex effluent, which includes E2 and EE2 from the contraceptive pill and hormone-replacement therapies [5]. While deleterious effects have been found on reproductive traits of individuals, Susan Jobling (Brunel University) showed that individual male reproductive success in breeding groups declines with increased feminization, but intersex fish can still breed [7]. Jobling's group has estimated modest increases in oestrogen equivalents in the UK's waterways over the time period to 2050 [8]. Thus, it seems pertinent to ascertain public opinion regarding intersex fish in European rivers and willingness to pay for water treatments that remove harmful levels of pharmaceuticals and other micro-pollutants from effluents.

Three Asian vulture species are now critically endangered because of acute toxicity following consumption of the carcases of diclofenac-treated livestock. Richard Cuthbert's (RSPB) work has been instrumental in banning the sale of diclofenac for veterinary use and promoting the sale of a vulture-safe alternative [6]. Consequently, there is an indication that endangered vulture populations are starting to recover, although numbers remain very low (less than 1% of previous levels) across South Asia. However, residues of diclofenac and other potentially toxic NSAIDs are still being detected in dead vultures.

## 3. Exposure pathways for wildlife

Recent improvements in analytical technologies have enabled the detection and quantification of pharmaceuticals in environmental matrices, including surface waters and soil [3]. However, efforts need to be targeted and measured concentrations presented within a risk-based context. Exposure risk can be estimated from prescription and sales figures, then refined based on the excretion of un-metabolized ‘parent compounds’ or bioactive metabolites, persistence in the environment and potential to bioaccumulate in food chains. Such an approach has been used by Chris Metcalfe (Trent University) to predict environmental concentrations of antidepressants. Long-term monitoring of pharmaceuticals in rivers and lakes confirmed the presence of antidepressants and other pharmaceuticals in plumes around STW outflows [9]. Wild fish caged within these plumes exhibited significant changes in a range of biomarkers, but consequences for individual fitness and population persistence are unknown.

Monitoring exposure to pharmaceuticals in terrestrial ecosystems is less well developed than in freshwater, but transferable techniques have been used to assess exposure risk to plant protection products on farmland [10]. Via radio-tracking, Helen Thompson (FERA) has mapped how wild birds and mammals disperse around contaminated resources at the landscape scale, which could also be used to record the movements of terrestrial vertebrates on STWs or sewage sludge fertilized fields, for example. At the national level, Richard Shore (CEH) suggested that current wildlife monitoring schemes, for example, the Predatory Bird Monitoring Scheme (http://pbms.ceh.ac.uk/), could be adapted to include surveillance for pharmaceutical exposure in addition to other contaminants [11]. Temporal and spatial trends in pharmaceutical exposure could therefore potentially be traced.

As with terrestrial habitats, there is a lack of data on pharmaceutical exposures for the marine environment, highlighted by Sally Gaw (University of Canterbury), despite it being a major receptor for wastewater due to increasing human habitation of coastal areas and more intensive use of pharmaceuticals in aquaculture.

## 4. Uptake and fate of pharmaceuticals in food webs

In contaminated environments, uptake of pharmaceuticals by invertebrates was shown by Alistair Boxall (University of York) to vary depending on the chemistry of the environmental matrix and species’ mode of feeding, thus effects can be difficult to predict [12]. Similarly, in terrestrial vertebrates, understanding consequences of group versus solitary feeding, or responses to novel food types, is important when designing captive experiments to calculate a realistic uptake rate [10]. Extending his work on the uptake and fate of NSAIDs in Asian vultures [6,13], Mark Taggart (University of the Highlands and Islands) is also analysing vulture and livestock samples with the aim of assessing the true risk of veterinary antibiotics to scavenging birds in Spain, a stronghold for vultures in Europe. Understanding the ecology of susceptible, exposed animals is vital.

## 5. Behaviour of pharmaceuticals

Risk assessment in the twenty-first century faces many challenges, including the growing numbers of compounds in circulation, while avoiding excessive use of animal testing. Thomas Hutchinson (CEFAS) described recent work on developing the OECD's Adverse Outcome Pathway (AOP) approach to prioritize species selection for laboratory research and field monitoring; it uses six levels of information ranging from the chemical properties of a toxicant through to population impacts [14]. While there are data gaps for pharmaceuticals, this AOP approach can facilitate read-across between chemicals with similar modes of action across diverse taxa. This can increase the power of such models to predict pharmaceutical effects on wildlife [4].

Judith Madden (LJMU) demonstrated the use and limitations of *in silico* tools for predicting ecotoxicity of pharmaceuticals, including predictions based on simple physico-chemical properties, as well as tools for grouping compounds into categories to allow for read-across. Categories can be formed using profilers for relevant interactions such as DNA or oestrogen receptor binding [15]. While models are also available for uptake and metabolism, understanding inter- and intraspecies variation is fundamental to predicting toxicity. Experiments by Vinny Naidoo (University of Pretoria) have shown that *Gyps* and other vulture species have an unusual metabolism with respect to NSAIDs [16]; they are suggested to be CYP 2C9 deficient or diminished, as are cats, which are also highly sensitive to NSAIDs and so may provide a pharmacokinetic model for vultures.

## 6. Effects of pharmaceuticals on wildlife

The paucity of studies on the effects of pharmaceuticals on non-model, particularly terrestrial, species was further highlighted by Judit Smits (University of Calgary). Essentially, we now need further development of non-lethal assays or biomarkers of subclinical toxicity following exposure to contaminants, for example, biotransformation enzymes and hormones [17]. Key features of an ideal sentinel for pharmaceutical risk in the wild include natural risk of exposure, toleration of human disturbance and relevance to the food web of interest. One such species is the European starling *Sturnus vulgaris*, which commonly feeds on pharmaceutical-contaminated invertebrates living on STWs. The species is robust to capture and captivity and forages on invertebrates, a potentially important but unstudied exposure route. Kathryn Arnold (University of York) found that long-term exposure to an environmentally relevant dosage of fluoxetine (Prozac), a commonly used antidepressant, altered physiology, behaviour and mass balance in starlings. While behaviours are non-lethal and ecologically relevant endpoints to measure in such studies, they can be challenging to analyse and interpret because of the high degree of individual variability [18].

Standardized laboratory tests and endpoints are required in order to ensure the reliability and repeatability of ecotoxicological studies. Exposure conditions and test organisms need careful consideration, as, for example, there may be significant inter- and intraspecies variation in sensitivity, requiring the use of additional safety factors. Although many studies claim to use outbred strains, which are considered more representative of wild populations, these claims are rarely supported by pedigree or genetic evaluation. Ross Brown (AstraZeneca and University of Exeter) has shown that inbred family lines of zebrafish can differ significantly in their physiological and developmental responses to pharmaceuticals, compared with outbred wild-type family lines [19].

## 7. Grand challenges for research, regulation and policy development

We identified several grand challenges for researchers in this field including; firstly, realistic assessments of exposure risks to pharmaceuticals, which are missing for most species, should account for the diversity and abundance of pharmaceuticals in environmental matrices, dispersal data for animals in contaminated landscapes and processes affecting uptake via diet or other routes. Assessments need to be future-proofed against increasing water scarcity and recycling of raw sewage, wastewater and application of sewage sludge in agriculture.

Secondly, current prospective risk assessments are based on individuals exposed to a single pharmaceutical under relatively benign laboratory conditions. In reality, animals are exposed to cocktails of chemicals, including pharmaceuticals and multiple environmental stressors, which can interact synergistically, additively or antagonistically [3]. Pharmaceutical impacts, where they occur, need to be distinguished from variation in fitness-related traits due to natural or anthropogenically mediated fluctuations in food availability, parasitism, etc.

Thirdly, for assessing pharmaceutical risks to wildlife globally, we need to focus on the developing world, where pharmaceutical production and consumption is rapidly increasing. With little or no treatment of some manufacturing discharges or municipal and agricultural waste streams containing human and veterinary pharmaceuticals, risks to wildlife and humans are predicted to be high, but remain virtually unassessed [20,21]. Such scientific research could support future international development policies.

Populations of many species living in human-altered landscapes are declining for reasons that often cannot be fully explained. Therefore, we believe that diverse approaches used by academic researchers, industry risk assessors and regulators need to be better integrated to assess current and future risks from pharmaceuticals in the environment.
